# Impedance‐Matched High‐Overtone Thickness‐Shear Bulk Acoustic Resonators With Scalable Mode Volume

**DOI:** 10.1002/advs.75456

**Published:** 2026-04-27

**Authors:** Zi‐Dong Zhang, Zhen‐Hui Qin, Yi‐Han He, Yun‐Fei Cheng, Hao Yan, Si‐Yuan Yu, Ming‐Hui Lu, Yan‐Feng Chen

**Affiliations:** ^1^ State Key Laboratory of Solid State Microstructures Department of Materials Science and Engineering Nanjing University Nanjing China; ^2^ Institute for Frontier Science Nanjing University of Aeronautics and Astronautics Nanjing China; ^3^ Collaborative Innovation Center of Advanced Microstructures Nanjing University Nanjing China; ^4^ Jiangsu Key Laboratory of Artificial Functional Materials Nanjing University Nanjing China

**Keywords:** high‐overtone bulk acoustic resonators, impedance‐matched, lithium niobate, microwave signal processing, thickness‐shear‐mode

## Abstract

High‐overtone bulk acoustic resonators (HBARs) are widely used in microwave signal processing due to their high quality factors and multimode resonances. However, conventional HBARs often suffer from impedance mismatch at metal interfaces, spurious modes, and limited flexibility in resonant mode volume design. Here we demonstrate a laterally excited high‐overtone thickness‐shear bulk acoustic resonator (X‐HTBAR) based on a 3 µm 128° Y‐cut LiNbO_3_ film on a high‐resistivity Si substrate, enabling excitation of thickness‐shear modes without a bottom electrode. The planar electrode configuration confines elastic energy within the electrode gap region and provides stable free spectral ranges. Experiments show comb‐like phonon spectra from 0.1–1.8 GHz with quality factors of 10^3^–10^5^ and frequency–quality products exceeding 10^1^ ^3^. Gridded electrodes suppress spurious modes, while the strong electromechanical coupling of 128° Y‐cut LiNbO_3_ enables tunable resonant mode volumes (0.008–0.064 mm^3^). These results demonstrate an alternative architecture for multimode acoustic resonators with potential applications in microwave and electro‐acoustic systems.

## Graphical abstract

A bottom‐electrode‐free high‐overtone bulk acoustic resonator is achieved by lateral excitation of antisymmetric thickness‐shear modes on a lithium‐niobate‐on‐silicon platform. Planar electrode geometry eliminates conventional loading along the resonance path and confines acoustic energy within the electrode gap. Acoustic energy injected into the silicon cavity forms high‐order standing phonons and dense comb‐like multimode spectra, enabling chip‐scale gigahertz acoustic resonators.

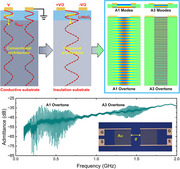

## 1 Introduction

High‐overtone bulk acoustic resonators (HBARs), as key components in microwave engineering, have been widely adopted in radio‐frequency signal processing [1, 2], low phase‐noise reference sources [3], and materials characterization systems [4, 5], owing to their high quality factors, broadband frequency coverage, and dense overtone spectra [6, 7, 8, 9, 10, 11, 12, 13]. In recent years, HBAR‐based resonators have also attracted attention for emerging applications such as microwave‐to‐optical conversion [14, 15, 16], hybrid electro‐acoustic systems [17, 18, 19, 20]. In these platforms, the dense multimode spectrum of HBARs provides opportunities for frequency‐multiplexed acoustic interactions and broadband signal processing.

**FIGURE 1:**
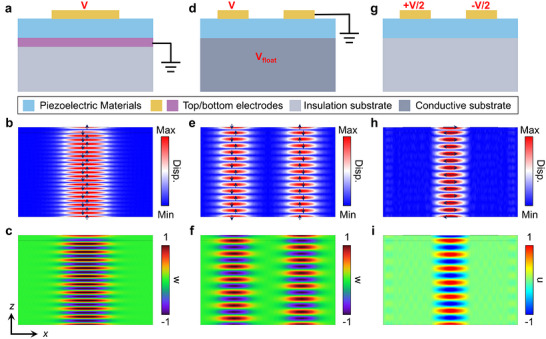
(a) Schematic of a conventional HBAR, comprising a metal/piezoelectric/metal transducer stack integrated with a low‐acoustic‐loss substrate. (b,c) Simulated total displacement field (b) and out‐of‐plane displacement field (c) at the resonant mode of a conventional HBAR. (d) Schematic of a single‐top‐electrode HBAR, wherein the conductive substrate simultaneously serves as the suspended bottom electrode and acoustic cavity. (e,f) Simulated total displacement field (e) and out‐of‐plane displacement field (f) at resonance for the single‐top‐electrode HBAR. (g) Schematic of a HTBAR consisting of an electrode/piezoelectric transducer on a low‐loss substrate. (h,i) Simulated total displacement field (h) and in‐plane displacement field (i) at the resonant mode of the HTBAR.

HBARs are a class of acoustic devices based on heterostructures, typically comprising a metal/piezoelectric film/metal sandwich‐type transducer integrated with a low‐loss substrate, as illustrated in Figure 1a. The transducer excites longitudinal acoustic waves via the inverse piezoelectric effect (see Figure 1b,c) and couples them into the underlying substrate. Acting as a Fabry–Pérot‐type acoustic cavity, the substrate reflects acoustic waves through impedance mismatch at its top and bottom surfaces, thereby forming stable standing wave modes and enabling high‐quality‐factor resonances along the longitudinal propagation direction. Multiple reflections of acoustic waves within the cavity give rise to high‐order overtone modes with narrow linewidths. The piezoelectric layer is commonly made from low‐dielectric‐ and low‐acoustic‐loss materials such as aluminum nitride (AlN) [12], scandium‐doped aluminum nitride (AlScN) [21], Gallium Nitride (GaN) [17], and barium strontium titanate (Ba_0.5_Sr_0.5_TiO_3_) [22]. To effectively minimize acoustic energy dissipation, substrates are typically selected from low‐acoustic‐loss materials such as diamond [21], sapphire [12], or silicon carbide (SiC) [6].

However, in conventional HBARs, the presence of a bottom metal electrode between the piezoelectric layer and the acoustic cavity disrupts the continuous acoustic impedance transition, thereby limiting efficient acoustic power transmission. This impedance mismatch induces fluctuations in mode spacing—i.e., instability in the free spectral range (FSR)—which is detrimental to applications such as information storage. Moreover, traditional HBARs are often designed with irregular geometries to suppress spurious vibration modes and parasitic effects such as stray capacitance and inductance [15, 17]. As a result, the resonant mode volume is relatively small and difficult to tailor, hindering their scalability and applicability in large‐scale integrated quantum and microwave photonic devices [16]. To improve impedance matching, superconducting niobium nitride (NbN) has been explored as a bottom electrode, significantly enhancing the acoustic coupling efficiency between the piezoelectric layer and the substrate [17]. Nevertheless, the superconducting properties of both the top and bottom electrodes rely on cryogenic conditions, limiting their practical deployment. To overcome these limitations, a novel single‐top‐metal‐electrode HBAR structure has been proposed (Figure 1d), in which a conductive substrate serves dually as the suspended bottom electrode and the acoustic cavity, thereby eliminating the need for a bottom metal layer [23, 24]. By judiciously selecting the material pairings—such as AlN or X‐cut LiNbO_3_ thin films on n‐type silicon carbide (SiC) substrates—superior acoustic impedance matching can be achieved, significantly reducing FSR fluctuations [24]. The transducer excites shear horizontal (SH) waves [23] or longitudinal acoustic waves [24] [See Supplementary Contents S2]. Nonetheless, phonon scattering at the metal‐piezoelectric (introduced by the top metal electrode) heterogeneous interface remains inevitable and continues to restrict device performance. In addition, this structure tends to cause spatial dispersion of acoustic energy, as illustrated in Figure 1e,f, where acoustic waves become localized under different electrodes. This leads to a reduction in acoustic energy injection efficiency. Moreover, the single‐top‐metal‐electrode HBAR requires a costly conductive substrate and still fails to enlarge the resonant mode volume, since the excited acoustic waves are confined between the top metal and bottom nonmetal electrodes (similar to those in conventional HBARs), making the mode volume dependent on the top electrode size.

In this study, we demonstrate a laterally excited HBAR (X‐HBAR) based on a LiNbO_3_‐on‐Si substrate, featuring improved acoustic impedance matching and scalable mode volume. Unlike conventional lateral overmoded bulk acoustic‐wave resonators (LOBARs), which rely on localized piezoelectric transducers to excite A0, S0, and S1 modes that propagate across a suspended substrate to form lateral overtones [25, 26, 27, 28], the X‐HBAR directly excites thickness shear modes via lateral electrodes without requiring bottom electrodes and confining the acoustic field between top electrodes, enabling high‐quality‐factor resonances along the longitudinal direction. The resonant region of the X‐HBAR comprises only the piezoelectric film and a low‐loss substrate, offering superior acoustic impedance matching and significantly reduced acoustic energy dissipation. Moreover, the lateral excitation scheme and the 128° Y‐cut LiNbO_3_, with its large piezoelectric coefficient and insensitivity to electrode spacing, enable a scalable resonant mode volume, thereby improving energy storage and coupling efficiency.

## 2 X‐HTBARs Structural Design and Working Mechanism

HTBARs consist of an electrode/piezoelectric transducer formed on a relatively thick, low‐loss substrate, as illustrated in Figure 1g. The piezoelectric layer employs a 3 µm‐thick 128° Y‐cut LiNbO_3_ thin film, while the substrate is a 500 µm‐thick (100)‐oriented high‐resistivity silicon (HR‐Si) wafer with a resistivity exceeding 10 000 Ω·cm. The 128° Y‐cut LiNbO_3_ features a large piezoelectric coefficient 𝑒_15_ (4.47C/m^2^) [29], enabling a high effective electromechanical coupling coefficient 𝑘^2^, thereby significantly enhancing the excitation efficiency of thickness‐shear modes. Importantly, the piezoelectric coefficients 𝑒_11_, 𝑒_12_, 𝑒_13_, and 𝑒_14_ are all zero in this crystal orientation, and the transverse shear coefficient 𝑒_16_ is relatively small (0.28 C/m^2^), effectively suppressing lateral spurious modes such as symmetric Lamb waves and their overtones. This results in enhanced spectral purity of the primary mode. The HR‐Si substrate offers multiple advantages: its high resistivity minimizes signal loss and parasitic capacitance, improving high‐frequency performance, signal isolation, and integrity; its excellent thermal conductivity supports efficient heat dissipation; and it is low‐cost and compatible with standard CMOS processes, offering outstanding process scalability. As shown in Figure 1h,i, the working principle of this structure is as follows: the transducer excites thickness‐shear modes via the inverse piezoelectric effect and couples the acoustic energy into the substrate. As further shown in Figure S1, the excitation of longitudinal acoustic waves is significantly weaker than that of thickness‐shear waves. Acting as a Fabry–Pérot‐type acoustic cavity, the HR‐Si substrate reflects acoustic waves through impedance mismatch at its top and bottom surfaces, thereby forming stable standing wave patterns and enabling narrow‐linewidth, high‐overtone resonances.

An idealized unattached piezoelectric transducer exhibits a spectral response that can be expressed as [29, 30, 31]

(1) $$f_{s}^{mn}=\sqrt{{\left(\frac{mv_{z}}{2t}\right)}^{2}+{\left(\frac{nv_{x}}{2p}\right)}^{2}}$$

where $f_{s}^{mn}$ denotes the resonant frequency of the (*m*, *n*) mode, *v_z_* and *v_x_* are the acoustic velocities along the shear and longitudinal directions, respectively. For lithium niobate, these values are 3592 and 6541 m/s, respectively [32]. The variables 𝑡 and 𝑝 refer to the thickness of the piezoelectric film and the period of the electrode structure, while 𝑚 and 𝑛 are the mode orders along the shear and longitudinal directions, respectively, taking integer values of 1, 2, 3, and so on. This study focuses on antisymmetric modes with a longitudinal mode order of 𝑛 = 1, and shear mode orders of 𝑚 = 1 and 𝑚 = 3. Under the condition that 𝑛 = 1 and the electrode pitch is much larger than the film thickness (i.e., 𝑡/𝑝 < 0.1), the resonant frequencies of the thickness‐shear modes can be approximated as

(2) $$f_{0}^{m1}\approx \frac{v_{z}}{2t}m.$$

**FIGURE 2:**
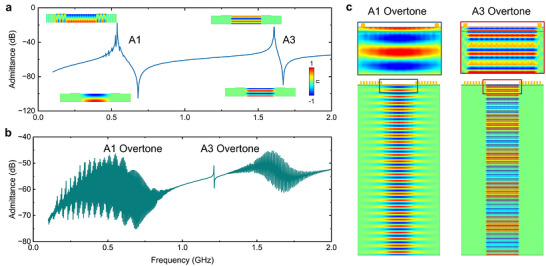
(a) Simulated admittance spectrum of an idealized unattached piezoelectric resonator. Insets illustrate the displacement mode profiles at the resonant and anti‐resonant frequencies for the A1 and A3 modes. (b) Simulated admittance spectrum of a laterally‐excited HTBAR. (c) Simulated displacement field profiles of the A1 and A3 overtone modes in the laterally‐excited HTBAR. The multi‐finger transducers are included in the simulations. The golden blocks on the top indicate the positions of the electrodes.

As indicated by Equation (2), for a given mode order, thinner piezoelectric films correspond to higher resonant frequencies. Taking the first‐order antisymmetric (A1) mode and third‐order antisymmetric (A3) mode as examples, their estimated resonant frequencies are approximately 0.598 and 1.796 GHz, respectively. Figure 2a shows the simulated admittance spectrum of the piezoelectric transducer. The insets illustrate the displacement mode profiles at both the resonant and anti‐resonant frequencies. The simulation results reveal that only odd‐order antisymmetric modes are effectively excited, with distinct resonant responses observed for the A1 and A3 modes at 0.54 and 1.609 GHz, respectively. The additional spectral features near the A1 resonance mainly originate from spurious acoustic modes, typically higher‐order A1 modes, arising from acoustic reflections and lateral standing‐wave formation within the resonator structure (See Supplementary Contents S9).

When the resonator is attached to a substrate with finite thickness, it exhibits a comb‐like phonon spectrum, as shown in Figure 2b, where a periodic frequency spacing between modes can be observed. A comb‐like phonon spectrum refers to a series of nearly equally spaced resonance peaks in the frequency domain, resembling the teeth of a comb. Figure 2b presents the simulated admittance curve of a HBAR, revealing two types of high overtones corresponding to the A1 and A3 modes. The simulated displacement mode shapes of the A1 and A3 overtones are shown in Figure 2c, confirming that the piezoelectric thin film excites the corresponding A1 and A3 modes and injects vibrational energy into the silicon substrate, thereby forming a high‐overtone bulk acoustic resonator. The resonance frequency of the *p*th‐order mode of the HBAR is given by

(3) $$f_{p}∼p\times \left(\frac{v_{\mathrm{si}}}{2t_{\mathrm{si}}}\right)$$

where *v*_si_ and *t*_si_ denote shear acoustic velocity and thickness of the substrate, respectively. The FSR between consecutive overtone modes is inversely proportional to the substrate thickness. For silicon, the acoustic velocities in the shear and longitudinal directions are 5846 and 8442 m/s, respectively [32]. When the silicon substrate thickness is 500 µm, the FSR is approximately 5.846 MHz. As shown in Figure S3 (a magnified view of Figure 2c), the FSR extracted from finite element simulations is about 5.8 MHz, which is in good agreement with the theoretical value calculated using Equation (3).

Acoustic impedance matching is crucial for efficient energy transfer from the piezoelectric layer to the acoustic cavity [7]. To minimize reflection losses and enhance energy transmission efficiency, the acoustic impedance of the piezoelectric layer must be matched to that of the substrate material. When a thickness‐shear mode wave propagates across the interface between two materials, the fractional reflected and transmitted power, denoted as *R* and *T*, respectively, can be calculated using Equation (4) [7].

(4) $$R={\left|\frac{Z_{1}-Z_{2}}{Z_{1}+Z_{2}}\right|}^{2},\;T=1-R$$

here, 𝑍 denotes the acoustic impedance of the material, defined as 𝑍 = 𝜌·𝑐, where 𝜌 and 𝑐 are the density and the shear acoustic velocity, respectively. The acoustic power transmission ratio from the piezoelectric layer (layer 0) to the 𝑖th layer is calculated by the following expression [7]

(5) $$P_{i}=P_{0}\times \prod_{1}^{i}T_{\left(i-1\right)\to i}.$$

where *P*_0_ denotes the generated acoustic power in the piezoelectric layer. The density of LiNbO_3_ is 4628 kg/m^3^, while that of silicon is 2329 kg/m^3^. Based on these values, the acoustic impedance for the thickness‐shear mode is calculated as 16.62 MRayls for lithium niobate and 13.62 MRayls for silicon, yielding an impedance ratio 𝑍_t_/𝑍_sub_ = 1.22. The good impedance matching between LiNbO_3_ and Si substrate enables efficient acoustic power transfer between them, exceeding 99%.

## 3 X‐HTBARs Performance Characterization

**FIGURE 3:**
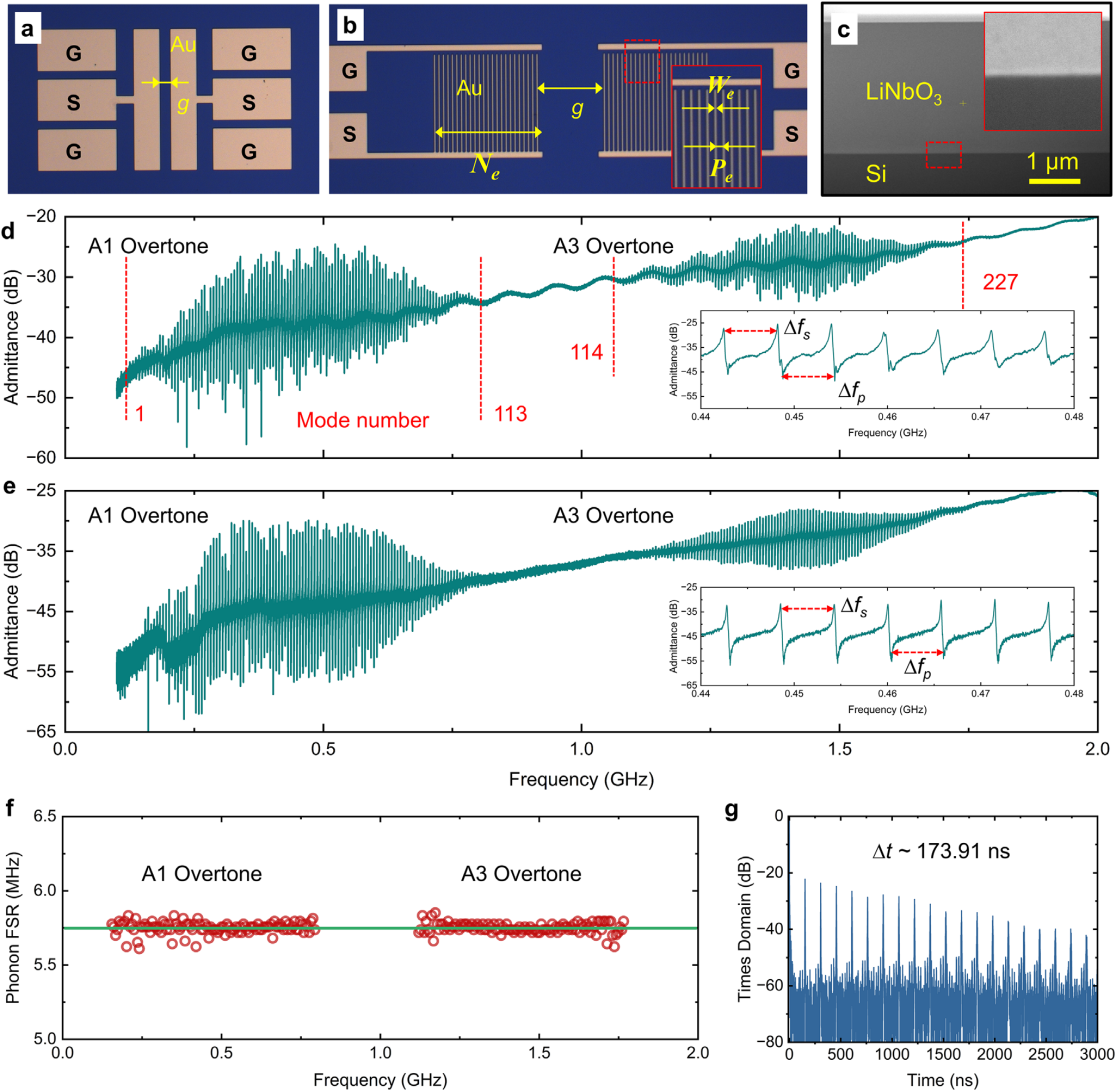
(a) Optical microscope images of an HTBAR featuring a two‐port single‐pair electrode configuration. (b) Optical microscope images of HTBARs with a multi‐pair electrode design. *W_e_*: electrode width, *N_e_*: Number of electrode pairs, *P_e_*: electrode pitch, *D_e_*: electrode duty cycle (*D_e_* = *W_e_*/(*P_e_* +*W_e_*)). (c) Cross‐sectional SEM image of the LN‐on‐Si. The inset presents a magnified SEM view of the interface. (d) Measured admittance spectrum of the single‐pair electrode device. Inset shows a magnified view highlighting the resonance peaks. The parameters Δ*f*_s_ and Δ*f*_p_ denote the frequency spacing between adjacent series and parallel resonance modes, respectively. (e) Measured admittance spectrum of the multi‐pair electrode device. Inset presents an enlarged view of the resonant features. (f) FSR of resonant modes for the laterally‐excited HTBARs with a multi‐pair electrode. (g) Time‐domain response of the laterally‐excited HTBARs obtained via fast Fourier transform (FFT) of the admittance spectrum.

We fabricated four types of X‐HTBARs with different electrode configurations, as illustrated in Figure S4a–d. Among them, the best‐performing devices are based on a two‐port single‐pair electrode structure and a grid‐electrode structure (Figure S4e–h), both equipped with rectangular ground–signal–ground (GSG) electrodes on either side. Their optical microscope images are shown in Figure 3a,b, respectively. A cross‐sectional scanning electron microscope (SEM) image of the LN‐on‐Si substrate is presented in Figure 3c, indicating a lithium niobate film thickness of 3.25 µm. Insets in Figure 3c and Figure S5a present magnified SEM images of the LiNbO_3–_silicon interface, confirming direct bonding of the LiNbO_3_ thin film to the silicon substrate. Figure S5b shows that the X‐ray rocking curve FWHM of the single‐crystal LiNbO_3_ is 0.0298°, indicating high crystalline quality. Figure S5c shows the AFM measurement of the LiNbO_3_ surface with a root mean square roughness of 0.207 nm, indicating an ultrasmooth surface with roughness well below ∼1 nm. On this heterogeneous substrate, excitation electrodes (200 nm Au/10 nm Cr) were fabricated using electron‐beam evaporation followed by photolithographic lift‐off, enabling the realization of the X‐HTBAR device. The frequency response of the X‐HTBAR was characterized in air at room temperature using a vector network analyzer. The admittance curve of the single‐pair electrode device is shown in Figure 3d. The measured results reveal a comb‐like phonon spectrum, with resonance modes primarily distributed in two frequency bands: 0.1–0.8 GHz and 1.1–1.8 GHz, corresponding to the A1 and A3 overtones, respectively. The A1 overtones include mode number 113, and the A_3_ overtones are associated with mode number 114. These experimental results are consistent with the numerical simulations presented in Figure 2. The discrepancy between the simulated and measured admittance values in the non‐resonant region mainly originates from parasitic electrical effects that are not fully included in the simplified simulation model. The admittance curve was obtained from a series of devices with varying electrode gaps, with the best‐performing device selected for presentation. The optimal device has an electrode gap 𝑔 of 100 µm, corresponding to a mode volume of 540 µm × 100 µm × 503 µm = 0.0272 mm^3^. This device exhibits high Q and minimal spurious modes, as highlighted in the magnified view in Figure 3d. Increasing the electrode area in conventional bulk acoustic resonators reduces resistance, improves power handling, and enhances energy confinement. Motivated by this, we widened the electrodes (Figure S6b), but spurious modes were not eliminated.

**FIGURE 4:**
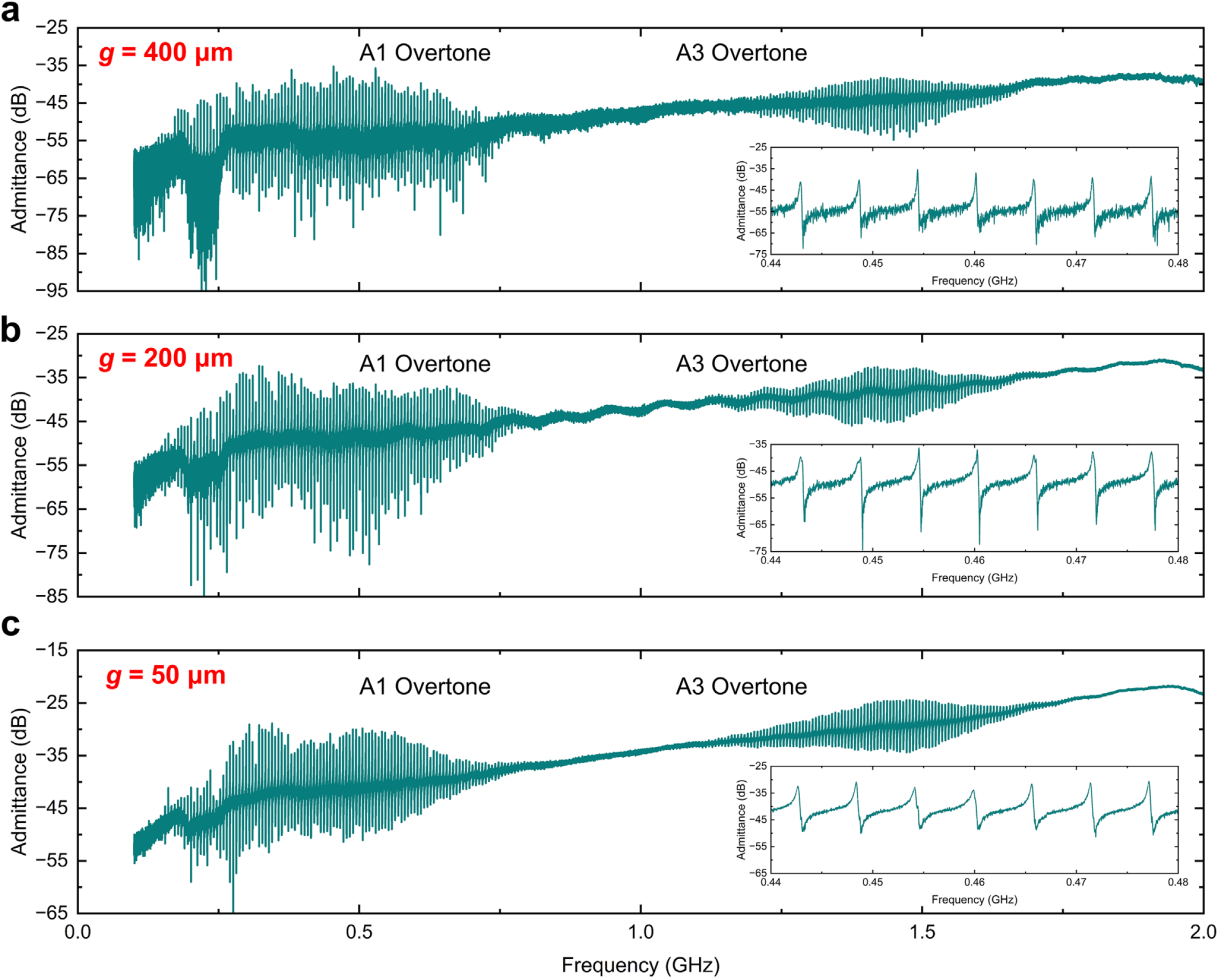
Measured admittance spectrum of the multi‐pair electrode device with different active region spacing 𝑔 for 400 µm (a), 200 µm (b), and 50 µm (c), respectively. Inset shows a magnified view highlighting the resonance peaks. As the gap size g varies, it primarily affects the insertion loss, with larger 𝑔 leading to higher insertion loss. Additionally, the Q of the resonator changes with 𝑔. When g equals 200 µm, the anti‐resonance Q reaches its maximum. However, across all values of 𝑔, no spurious modes appear in the resonance spectra.

To mitigate these spurious modes, we adopted a grid electrode design, in which a large electrode is divided into multiple small segments interconnected by thin busbars, as shown in Figure 3b. The segmentation breaks the acoustic continuity of a large electrode, effectively suppressing unwanted lateral vibrations. A higher number of grid‐electrode pairs increases Q and suppresses spurious modes, whereas smaller duty cycles and narrower electrode gaps further enhance resonator performance. A more detailed explanation is provided in Supplementary Contents S4. The ground bus lines are configured to ensure a uniform distribution of the excited acoustic waves across the electrodes, as shown in Figure S7. Taking the above factors into account, the electrode parameters were determined to be *W_e_* = 6 µm, *P_e_* = 10 µm, *D_e_* = 37.5%, and *N_e_* = 24. Furthermore, owing to the properties of 128° Y‐cut lithium niobate, which features a large piezoelectric stress constant (*e_15_*) and renders thickness‐shear resonators relatively insensitive to electrode spacing, increasing the active region spacing (𝑔) has only a minor effect on the effective electromechanical coupling coefficient while helping to suppress parasitic modes, as shown in Figure S8. Consequently, the proposed HBARs achieve tunable mode volumes while maintaining stable device performance even at large volumes. The admittance curve of the multi‐pair electrode device is shown in Figure 3d, exhibiting a comb‐like phonon spectrum primarily distributed over two frequency bands: 0.1–0.8 GHz and 1.1–1.8 GHz. Compared to the single‐pair electrode device, this configuration shows virtually no spurious modes across the entire operating bandwidth, as highlighted in the magnified view in Figure 3d. The mode volume of this device is 320 µm × 100 µm × 503 µm = 0.0161 mm^3^. Moreover, the active region spacing 𝑔 can be tuned from 50 to 400 µm (corresponding to mode volumes ranging from 0.008 to 0.064 mm^3^), with all resulting resonance states exhibiting high performance and only a few showing minor parasitic mode perturbations, as shown in Figure 4a–c. This demonstrates the excellent scalability of the modal volume in this configuration. The tunable mode volume reflects the geometric flexibility of the proposed device architecture.

Variations in the FSR offer an effective metric for assessing the acoustic impedance matching between different material layers in HBARs. We extracted the FSR of resonant modes from the device using the relation Δ𝑓 = 𝑓_p+1_−𝑓_p_ (Figure 3f). In the current structure, both the A1 and A3 mode resonances exhibit FSRs of approximately 5.75 MHz, which agrees well with the theoretical prediction from Equation (3). Statistical analysis further reveals that the average FSR (𝜇_FSR_) for the A1 mode is 5.75 MHz with a standard deviation (𝜎_FSR_) of 0.03906 MHz, yielding a normalized FSR variation factor 𝐶_FSR_ = 𝜎_FSR_/𝜇_FSR_ = 0.00627, which reflects the uniformity of mode spacing. For the A3 mode, the mean FSR is also 5.75 MHz, with a standard deviation of 0.033166 MHz and a corresponding 𝐶_FSR_ of 0.00577. While these 𝐶_FSR_ values are higher than those achieved in some state‐of‐the‐art longitudinal‐mode HBARs based on substrates grown by epitaxial processes [21, 24], they are expected to decrease significantly by replacing the single‐side polished silicon substrate with a double‐side polished one or through further optimization of the material stack and device design. Longitudinal‐mode HBARs have achieved extremely low 𝐶_FSR_ after decades of development, and we believe that the proposed X‐HTBARs will similarly attain high performance with continued refinement. The slight variation in FSR endows the HBAR device with the potential to realize a mechanical overtone frequency comb [33]. By performing a fast Fourier transform (FFT) on the admittance curve, the time‐domain transmission response of the X‐HTBAR was obtained, as shown in Figure 3g. A periodic pulse sequence with a time interval of approximately Δ𝑡 ≈ 137.91 ns was observed, corresponding to the round‐trip travel time of acoustic phonons within the substrate. It is worth noting that the FSR and Δ𝑡 are inversely related, following Δ𝑡 = (Δ𝑓)^−1^.

## 4 Quality Factors, Phonon Relaxation Time, and Temperature Stability

**FIGURE 5:**
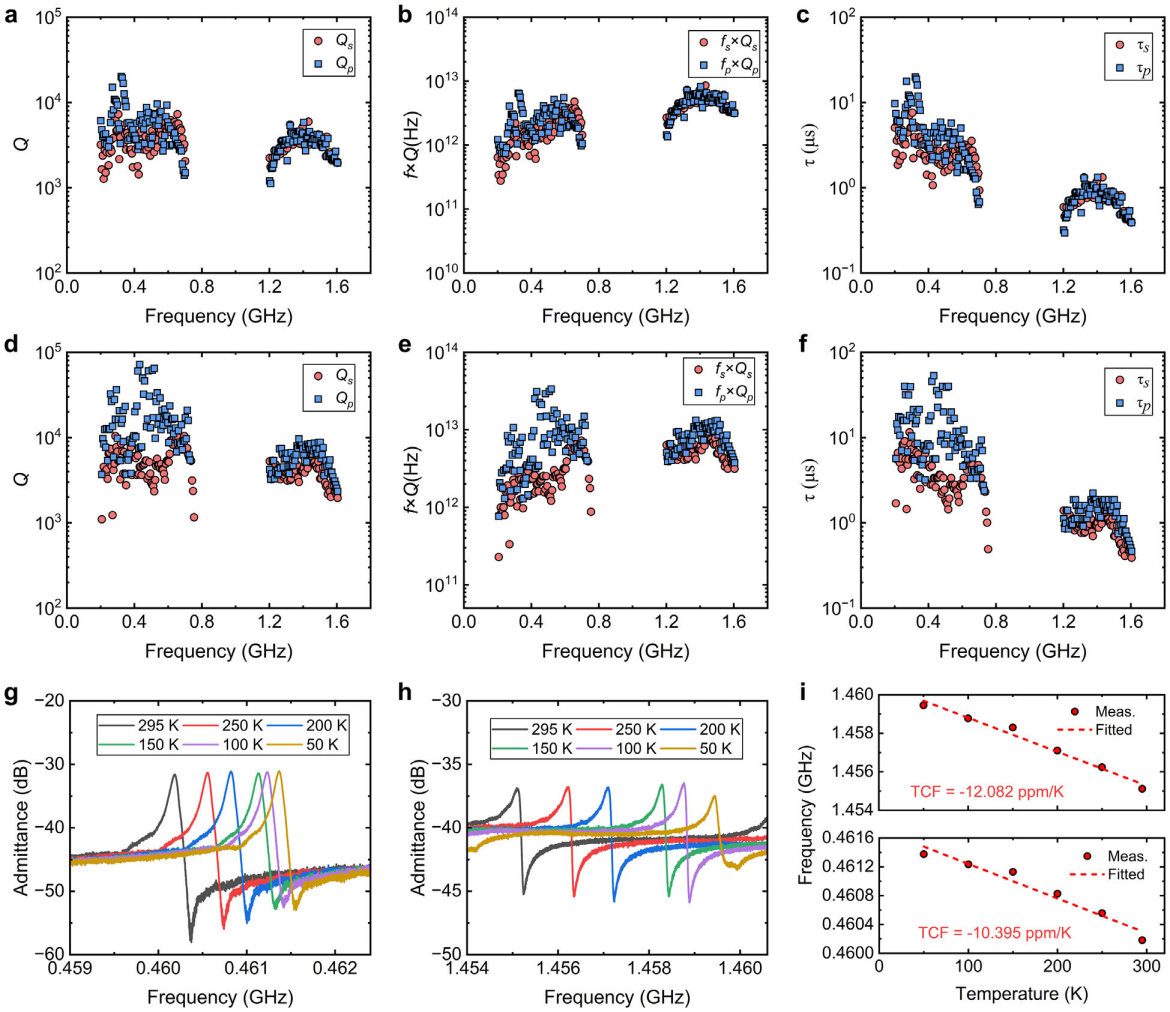
(a,d) Measured mechanical *Q* for the laterally‐excited HTBAR with a multi‐pair electrode configuration, for electrode gaps of 100 µm (a) and 200 µm (d). *Q_s_* is the resonance *Q*, and *Q_p_* is the anti‐resonance *Q*. (b,e) *f* × *Q* products for 100 µm (b) and 200 µm (e) gaps, which extracted from the same device. (c,f) Extracted phonon relaxation times (τ) for 100 µm (c) and 200 µm (f) gaps of the measured laterally‐excited HTBAR. These values were obtained under ambient temperature and pressure conditions. (g,h) Admittance spectra of the X‐HTBAR measured at various temperatures from 50 to 295 K, corresponding to the (d) A1‐mode overtone and (e) A3‐mode overtone, respectively. (i) Temperature‐dependent resonance frequencies and the extracted TCF.

The *Q* extracted from the measured data for electrode gaps of 100 µm are shown in Figure 5a. All resonant modes exhibit *Q* values exceeding 10^3^, with some modes reaching values above 10^4^ near 0.38 GHz. Given the frequency‐dependent variation of *Q*, the product of frequency and quality factor (*f* × *Q*) is widely adopted as a key figure of merit for evaluating resonator performance, as depicted in Figure 5b. Most resonant modes exhibit *f* × *Q* products exceeding 10^1^ ^2^. The *Q* factor can be further tuned by adjusting the spacing 𝑔 of the active region, as illustrated in Figure 4. Figure 5d,e shows the 𝑄 and 𝑓 × 𝑄 values for the device with an electrode gap of 200 µm, which are significantly enhanced compared with those of the 100 µm‐gap device. Notably, the A1 and A3 overtone modes achieve peak Q of 7.19 × 10^4^ and 9.65 × 10^3^ at 0.432 and 1.375 GHz, respectively. and *f* × *Q* values of 3.35 × 10^1^ ^3^ and 1.33 × 10^1^ ^3^ at 0.517 and 1.375 GHz, respectively. Although these values remain below those reported for some longitudinal‐mode HBARs, further improvements may be possible through device optimization, such as using low acoustic‐loss substrates, including sapphire or silicon carbide. As shown in Table S1, the acoustic impedances corresponding to the shear velocities of these two materials are also well matched with that of LN. Moreover, the phonon relaxation time, calculated as τ = 2𝑄/ω_𝑚_, is shown in Figure 5c, with values reaching approximately 19.9 µs. The 200 µm‐gap device exhibits a maximum phonon relaxation time of 53.04 µs (Figure 5f).

To assess the temperature stability of the X‐HTBARs, we measured their admittance spectra across a wide temperature range (50–295 K). Figure 5g,h displays the results for the A1‐mode and A3‐mode overtones, respectively. The temperature evolution of representative phonon modes—mode #54 near 0.46 GHz (Figure 5g) and mode #172 near 1.455 GHz (Figure 5h)—shows an increase in resonance frequency with decreasing temperature (T), due to the substrate's rising shear modulus and the consequent increase in shear sound velocity. Extended data on the Q_s_ and Q_p_ trends across multiple overtones are presented in Figure S9. The resonance Q_s_ remains relatively stable with decreasing temperature but shows a decline at lower temperatures, whereas the anti‐resonance Q_p_ continuously decreases with decreasing temperature. This is attributed to the significant mismatch in the coefficient of thermal expansion (CTE) between Si and LiNbO_3_, which induces substantial thermal stress in the LiNbO_3_ layer—especially across large temperature variations such as from room temperature to cryogenic conditions or during high‐temperature reflow processes. This issue can be mitigated by: (1) introducing CTE‐matched or graded buffer layers (e.g., SiO_2_, amorphous silicon) [34, 35]; (2) reducing the LiNbO_3_ layer thickness (e.g., < 1 µm) to lower thermal stress and enhance flexibility; (3) replacing hydrophilic direct bonding with surface‐activated bonding to minimize residual stress from high‐temperature processes. As shown in Supplementary Contents S8, a SiO_2_ compensation layer can be introduced between the LiNbO_3_ layer and the Si substrate. Although inserting a SiO_2_ compensation layer may introduce acoustic impedance mismatch and associated losses, when its thickness is kept below the acoustic wavelength, the impact on resonator performance is generally limited, mainly resulting in a shift of the resonance frequency. Therefore, the temperature dependence of the *Q* factor is not discussed in detail here. Figure 5i shows the fitted temperature dependence of the resonance frequencies for mode #54 and mode #172, corresponding to the A1‐mode and A3‐mode overtones, respectively. The extracted TCF are approximately −10.395 ppm/K for the A1‐mode and −12.082 ppm/K for the A3‐mode, which are comparable to those of other LiNbO_3_‐based HBARs [23] and significantly lower than most previously reported A1 resonators, likely due to the solidly mounted configuration [36].

Although the measured performance remains below that of some recently reported HBAR platforms, as summarized in Table 1, this work proposes a different X‐HTBAR architecture, which enables overtone excitation through the A1 and A3 modes without requiring a bottom electrode and allows tunable acoustic mode volumes. In M/X‐cut LN/n‐SiC HBARs [23], the excited shear‐horizontal (SH) mode, belonging to the class of thickness‐shear resonances with displacement orthogonal to the electric field, is fundamentally different from the thickness‐shear mode studied here, featuring displacement largely aligned with the electric field and thus enabling a different effective electromechanical driving component. And similar *Q* factors, *f* × *Q* products, and *C_FSR_* values were obtained even for relatively large mode volumes, demonstrating the feasibility of the proposed architecture. Due to material availability, the experiments were conducted on single‐side polished wafers, whose non‐uniform backside can introduce phonon diffraction and may affect device performance. HR‐Si preserves the mechanical robustness and fabrication compatibility of standard silicon, while offering significantly lower cost compared to other low‐loss substrates such as SiC, sapphire, and diamond. The LiNbO_3_‐on‐Si platform is compatible with silicon fabrication processes and may provide a useful platform for multimode acoustic resonators and frequency‐multiplexed microwave acoustic devices.

**TABLE 1:** Comparison of Existing HBARs Configurations.

Material stack	Acoustic mode	Bottom electrode	Excitation region	Cost	*fQ* (10^13^ Hz)	C_FSR_	Mode V^g^ (mm^3^)	Refs.
M^a^/GaN/NbN/SiC	L‐mode^b^	Yes, NbN	Top–bot^e^	High	230	8.2 × 10^−4^	0.003	[17]
M/AlN/n‐SiC	L‐mode	Yes, n‐SiC	Top–bot	Medium	1.3	4.8 × 10^−4^	0.00023	[24]
M/X‐cut LN/n‐SiC	SH‐mode^c^	Yes, n‐SiC	Top–bot	Medium	9.6		0.0004	[23]
M/BSTO/M/sapphire	L‐mode	Yes, M	Top–bot	Medium	6	3.1 × 10^−3^	0.003	[5]
M/AlScN/M/diamond	L‐mode	Yes, M	Top–bot	High	40	2.6 × 10^−3^	0.00023∼0.0049	[21]
ε‐Ga2O3/n‐SiC	L‐mode	Yes, n‐SiC	Top–bot	Medium	12	7.8 × 10^−3^	0.009	[41]
2DEG^h^/GaN/SiC	L‐mode	Yes, 2DEG	Top–bot	Medium	1.7		0.0028	[11]
M/ScAlN/NbN/SiC	L‐mode	Yes, NbN	Top–bot	High	10		0.0014	[13]
M/AlScN/M/Si	L‐mode	Yes, M	Top–bot	low	0.22		0.065	[42]
M/AlGaN/2DEG/GaN/AlN/SiC	L‐mode	Yes, 2DEG	Top–bot	High	10		0.009	[6]
M/X‐cut LN/n‐SiC	SH‐mode	Yes, n‐SiC	Top–bot	Medium	3.2		0.003	[43]
M/128°Y‐cut LN/HR‐Si	**TS‐mode** ^d^ **(*A1, A3*)**	**No**	**Top‐Top** ^f^	**low**	**3.35 (A1)**	**6.27 × 10^−3^**	**0.008∼ 0.064**	**Ours**
					**1.33 (A3)**	**5.7 × 10^−3^**		**Ours**

^a^ M: metal.

^b^ L‐mode: longitudinal‐mode (thickness‐expansion bulk mode).

^c^ SH‐mode: shear‐horizontal mode, being a type of thickness‐shear mode.

^d^ TS‐mode: thickness‐shear‐mode.

^e^ Top–bot: Acoustic wave excited between top and bottom electrodes.

^f^ Top‐Top: Acoustic wave excited between top electrodes.

^g^ V: volume.

^h^ DEG: two‐dimensional electron gas.

## 5 Conclusions

This study demonstrates a X‐HTBAR based on a LiNbO_3_‐on‐Si platform. By employing a 128° Y‐cut LiNbO_3_ film and a lateral excitation scheme without a bottom electrode, the device enables effective excitation of thickness‐shear modes while improving acoustic impedance continuity between the piezoelectric layer and the substrate. The gridded electrode design helps suppress spurious modes and allows scalable resonant mode volumes. Experimental measurements show stable high‐quality‐factor resonances with frequency–quality factor products exceeding 10^1^ ^3^, consistent free spectral ranges, scalable mode volumes, and low temperature coefficients. The strong electromechanical coupling of 128° Y‐cut LiNbO_3_ also makes the device relatively insensitive to electrode spacing while confining the acoustic field primarily within the electrode gap region. Rather than aiming to maximize the ultimate performance limits of HBAR devices, this work demonstrates an alternative architecture that may complement existing HBAR and XBAR (Cross‐sectional Bulk Acoustic Resonator) platforms for multimode acoustic resonators and microwave‐frequency signal processing [37, 38]. The LiNbO_3_‐on‐Si platform is compatible with silicon‐based fabrication processes and may facilitate integration with microwave acoustic and microwave photonic systems [39, 40]. Further optimization of substrate materials and acoustic confinement structures, such as low‐loss substrates including sapphire or silicon carbide, may enable improved device performance and broaden the potential applications of this platform.

## 6 Methods

### 6.1 Simulations of the HBAR with Different Configurations

Finite element analysis is carried out using the Piezoelectric Devices Module in COMSOL Multiphysics. We model the spectral response of an idealized lithium niobate piezoelectric transducer suspended without attachment to a substrate, as illustrated in Figure 2a. This essentially represents a film bulk acoustic resonator with free‐free mechanical boundary conditions. This is intended to verify the operating mechanism of the X‐HTBARs. In the simulations of different types of HBARs, the electrical configurations are set according to Figure 1a,d,g. The top and bottom boundaries are defined as free boundaries, while the left and right boundaries are set as low‐reflection boundaries. In the simulations, energy dissipation is taken into account by incorporating mechanical damping (isotropic loss factor of 0.001) and dielectric loss (isotropic dielectric loss factor of 0.005). The material parameters are taken from the COMSOL material library. During meshing, the maximum element size is set to approximately one‐tenth of the wavelength. In the frequency‐domain simulation, the V_in_ terminal is defined as a power excitation port with an input power of P = 1 W, while the V_out_ terminal is defined as a terminated port with P = 0 W. Under these boundary conditions, the electric potential and electric field distribution are solved self‐consistently under the applied excitation.

### 6.2 Device Fabrication and Characterization

The X‐HTBARs device was manufactured using a 128° Y‐cut lithium niobate‐on‐silicon substrate. The LN‐on‐Si substrate was custom ordered from Jingzheng Corporation (NanoLN), China. First, the LiNbO_3_ was directly bonded onto a HR‐Si substrate, followed by polishing the LiNbO_3_ layer down to 3 µm. Due to fabrication variations, the actual LiNbO_3_ thickness used in this work is 3.25 µm. Note: Due to material availability, the experiments were performed on single‐side polished wafers. Next, the metal electrodes were formed through e‐beam evaporation metal and then the lift‐off process. The electrode structure consisted of a bilayer configuration (10 nm Cr/200 nm Au). Radiofrequency characterization involved collecting two‐port scattering parameters under standard ambient conditions using a calibrated vector network analyzer (Agilent M9005A, 10 MHz–26 GHz range). Electrical contact establishment employed TITAN T26A‐GSG0200 probes featuring 200 µm spacing between Ground‐Signal‐Ground contacts, with the device under test positioned on a probe station platform. The input power level from the network analyzer was maintained at −5 dBm during all measurements. The S parameter test under vacuum and cryogenic temperature is realized by a closed‐cycle vacuum cryogenic temperature probe station (Inplatinum Scientific Instruments (Shanghai) Co., Ltd.‐CPS‐50) connected with vector network analyzer (Agilent M9005A, 10 MHz–26 GHz range), where the room temperature vacuum is 10^−4^ mbar, and the cryogenic temperature vacuum is about 5 × 10^−6^ mbar. Standard RF calibration (OPEN, SHORT, LOAD, and THRU) was performed on the probe station prior to measurements.

## Conflicts of Interest

The authors declare no conflicts of interest.

## Supporting information




**Supporting File**: advs75456‐sup‐0001‐SuppMat.docx.

## Data Availability

The data that support the findings of this study are available from the corresponding author upon reasonable request.
