# Supplementary material for: Impedance‐Matched High‐Overtone Thickness‐Shear Bulk Acoustic Resonators With Scalable Mode Volume
Source: Adv Sci (Weinh). 2026 Apr 27;13(41):e75456. doi: 10.1002/advs.75456 (PMC13335547; doi:10.1002/advs.75456)
Supplement: Supplementary file 1 — Supporting File: advs75456‐sup‐0001‐SuppMat.docx. [file ADVS-13-e75456-s001.docx]

**Supplementary materials**

**Impedance-matched High-Overtone Thickness-Shear Bulk Acoustic Resonators with Scalable Mode Volume**

Zi-Dong Zhang^1,2 *^, Zhen-Hui Qin^1^, Yi-Han He^1^, Yun-Fei Cheng^1^, Hao Yan^1^, Si-Yuan Yu^1,3,4^, Ming-Hui Lu^1,3,4 †^, and Yan-Feng Chen^1,3^

^1^ State Key Laboratory of Solid State Microstructures, Department of Materials Science and Engineering, Nanjing University, Nanjing 210093, China

^2^ Institute for Frontier Science, Nanjing University of Aeronautics and Astronautics, Nanjing 210016, China

^3^ Collaborative Innovation Center of Advanced Microstructures, Nanjing University, Nanjing 210093, China

^4^ Jiangsu Key Laboratory of Artificial Functional Materials, Nanjing University, Nanjing 210093, China

Email: [zdzhang@nuaa.edu.cn](mailto:zdzhang@nuaa.edu.cn) (*Zi-Dong Zhang*); [luminghui@nju.edu.cn](mailto:luminghui@nju.edu.cn) (*Ming-Hui Lu*)

**CONTENTS**

1. Comparison of the characteristics of acoustic waves from different types of HBARs

2. Compared with HBARs realized on X-cut LN/n-SiC

3. Comparison of Device Performance for X-HTBARs with Various Electrode Designs

4. Influence of electrode structural parameters on device performance

5. Effects of different ground bus configurations on acoustic waves and electric fields

6. The reason for the device's tunable mode volume

7. CTE Mismatch Reduces *Q_p_* and *Q_s_* in Multiple Overtones

8. Compensation Layer to Mitigate CTE Mismatch

9. Origin of spurious spectral features near the A1 resonance in Fig. 2a

**1. Comparison of the characteristics of acoustic waves from different types of HBARs**

Figures S1a–c show the displacement field distributions corresponding to the resonant peaks of a conventional HBAR. The overall displacement field is dominated by the out-of-plane component, while the in-plane displacement is about an order of magnitude smaller. Figures S1d–f present the displacement field distributions of a single-top-electrode HBAR. Similarly, the overall displacement field is governed by the out-of-plane component, with the in-plane displacement being an order of magnitude weaker. However, the use of a GSG interface for excitation leads to energy partitioning beneath different electrodes. Figure S1g–i illustrate the displacement field distributions of the HTBAR proposed in this work. In contrast to the previous two cases, the overall displacement field here is primarily determined by the in-plane component, while the out-of-plane displacement is about an order of magnitude smaller.

**Fig. S1.** Comparison of the dominant acoustic wave types in HBARs with different configurations. a-c) Simulated total displacement field (a), out-of-plane displacement field (b) and in-plane displacement field (c) at the resonant mode of a conventional HBAR. d-f) Simulated total displacement field (d), out-of-plane displacement field (e) and in-plane displacement field (f) at the resonant mode of a single-top-electrode HBAR. g-i) Simulated total displacement field (g), out-of-plane displacement field (h) and in-plane displacement field (i) at the resonant mode of the HTBAR. The excitation of longitudinal acoustic waves is significantly weaker than that of thickness-shear waves for the HTBAR; however, in a conventional HBAR and a single-top-electrode HBAR, longitudinal acoustic waves are predominantly excited.

**2. Compared with HBARs realized on X-cut LN/n-SiC**

**Our device excites thickness-shear modes, namely the A1 and A3 modes. Excitation of** **thickness-shear modes requires LiNbO_3_ with** **a large 𝑒_15_ piezoelectric stress constant, which is typically achieved with Z-cut (3.7 C·m⁻^2^) or** **128° Y-cut LiNbO₃ (4.47 C·m⁻^2^).** **In contrast, X-cut LiNbO_3_ has a negligible 𝑒_15_ (~0 C·m⁻²), but it instead exhibits a significant 𝑒_16_ (–3.7 C·m⁻²), making it suitable for exciting** **shear horizontal (SH) waves. Since thickness-shear modes are excited, our structure requires no bottom electrode, and places the excited bulk mode between electrodes rather than directly beneath them. This fundamentally differs from conventional designs and readily facilitates interaction with other excitations, such as optical fields.**

**Simulations using Ref. 1 parameters [**Fig. S2**] confirm that without a bottom electrode, the SH mode cannot be excited** [Fig. S2c]**.** Even with a bottom electrode, the SH mode can be excited; however, many parasitic modes are present, as shown in Fig. S2d, which ultimately causes conventional HBARs to exhibit numerous spurious modes.

**Fig. S2.** Simulations of X-cut LiNbO_3_ thin-film resonators using the parameters from Ref. 2. (a, b) Schematic of the electrode structures with X-cut LiNbO_3_, a) without a bottom electrode, b) with a bottom electrode. c, d) Simulated conductance responses of the resonator with c) and without d) a bottom electrode. The inset in d) shows the excited vibration modes at 1.61 GHz and 3.16 GHz, respectively.

**Fig. S3.** A magnified view of Fig. 2c, with a frequency range from 0.35 GHz to 0.38 GHz. The FSR is approximately 5.8 MHz.

**Table S1: Comparison of substrate properties.**

|  | Mass density  (kg/m^3^) | Vertical Velocity  (m/s) | Acoustic impedance  (MRayls) | Transmitted power | Reference |
| --- | --- | --- | --- | --- | --- |
| LiNbO_3_ | 4628 | 3592 | 16.624 | 1 | 2, 3 |
| Silicon | 2329 | 5846 | 13.615 | 0.990 | 4, 5 |
| 3C-SiC | 3210 | 8501 | 27.288 | 0.940 | 6, 7 |
| 4H-SiC | 3210 | 7126 | 22.874 | 0.975 | 8, 9 |
| 6H-SiC | 3210 | 7126 | 22.874 | 0.975 | 8, 9 |
| Diamond | 3515 | 12823 | 45.072 | 0.787 | 10, 11 |
| Sapphire | 3968 | 6045 | 23.986 | 0.967 | 12, 13 |
| α-quartz | 2649 | 4675 | 12.384 | 0.979 | 14, 15 |
| SiO_2_ | 2200 | 3198 | 7.035 | 0.836 | 16, 17 |

**3. Comparison of Device Performance for X-HTBARs with Various Electrode Designs**

We fabricated four types of X-HTBARs with different electrode configurations—namely, a single-port single-electrode, a dual-port ring-electrode, a two-port single-pair-electrode, and a grid-electrode design—as shown in Figs. S4a–d. Among these, the best-performing devices are based on the two-port single-pair and grid-electrode electrode structures (Figs. S4e–h), exhibiting higher impedance ratios and both featuring rectangular ground–signal–ground (GSG) electrodes on either side. While devices with single-port single-electrode and dual-port ring-electrode configurations show impedance ratios below 10 dB, the two-port single-pair and grid-electrode devices achieve maximum impedance ratios approaching 30 dB.

**Fig. S4.** Device structures and performances of four types of X-HTBARs with different electrode configurations. a-d) Optical microscope images of an HTBAR featuring a single-port single electrode configuration (a), a dual-port ring electrode configuration (b), a two-port single-pair electrode configuration (c) and a grid-electrode configuration (d), respectively. The device dimensions are labeled in the figure. e-h) Measured admittance spectrum corresponding to the device structures in Figs. a–d, respectively. Among them, the best-performing devices are based on a two-port single-pair electrode structure and a multi-pair electrode structure, and other structures have lower impedance ratios.

**Fig. S5.** (a) Magnified SEM image of the LiNbO_3_ thin film–Si substrate interface. (b) X-ray rocking curve of single-crystal LiNbO_3_ (FWHM = 0.0298°). (c) AFM measurement of the LiNbO₃ surface. The polished single-crystal LiNbO_3_ surface exhibits a root-mean-square roughness (Ra) of 0.207 nm as measured by AFM, indicating an ultrasmooth surface with roughness well below ~1 nm.

**4. Influence of electrode structural parameters on device performance**

To illustrate the selection rules for the "grid electrodes" parameters, a 785 MHz resonant mode is chosen as a representative case to detail the electrode configuration. As shown in Fig. S6b, the resonance curve of a single electrode with a width of 6 μm exhibits a high-order spurious mode (see Fig. S6g). From conventional bulk acoustic resonators, it is known that increasing the electrode area reduces resistance, enhances power handling capability, and improves energy confinement, thereby benefiting both the quality factor (*Q*) and power capacity. Motivated by this, we increased the electrode area (Fig. S6b), achieved by widening the electrodes, as shown in Fig. S6a. The results show that while *Q* indeed improves, the spurious modes remain significant.

To mitigate these spurious modes, we adopted **a grid electrode design**, in which a large electrode is divided into multiple small segments interconnected by thin busbars. This approach provides several critical advantages:

- Reduced static capacitance (*C_0_*): Compared with a solid electrode, grid segmentation greatly lowers *C_0_*, thereby increasing bandwidth and impedance. Since *C_0_* mainly depends on the overlapping electrode area, only the small facing segments contribute significantly, while the contribution from the narrow interconnections is negligible.
- Suppression of lateral modes: The segmentation breaks the acoustic continuity of a large electrode, effectively suppressing unwanted lateral vibrations and concentrating energy in the main mode, leading to cleaner and sharper filter responses.

However, excessively enlarging the electrode area inevitably increases *C_0_*, which reduces the effective electromechanical coupling coefficient, narrows the bandwidth, enlarges chip size, and shifts the resonant frequency. Thus, careful parameter optimization is required.

The number, spacing, and width of the grid electrodes were optimized based on both simulation and experimental results. Increasing the number of grid-electrode pairs improves the quality factor (*Q*) and helps suppress spurious modes, but the improvement saturates beyond a certain point (Fig. S6c). The duty cycle also affects performance: smaller duty cycles lead to higher *Q* (Fig. S6d). Electrode spacing influences both the resonance and energy confinement: narrower gaps enhance resonator performance (Fig. S6e). Overall, these parameters were chosen to balance *Q*, effective electromechanical coupling (*k^2^*), suppression of spurious modes, and device footprint, reflecting the underlying physics that governs energy confinement and acoustic field uniformity.

**Fig. S6.** Influence of electrode structural parameters on device performance. a) Optical microscope images of HTBARs with a multi-pair electrode design. b–e) Effects of electrode width (b), electrode pair number (c), electrode duty cycle (d), and electrode spacing (e) on device performance. f) Vibration mode of the target A1 overtone modes. g) Vibration mode of higher-order spurious modes.

**5.** **Effects of different ground bus configurations on** **acoustic waves and electric fields**

The ground bus lines are configured to ensure a uniform distribution of the excited acoustic waves across the electrodes, as shown in Fig. S7. When no ground bus lines are present (Figs. S7b, e, h), the resulting acoustic field is uneven, with waves biased toward the excitation bus. To mitigate this effect, ground electrodes can be added on both sides of the grid electrodes (Figs. S7c, f, i), which improves uniformity between the electrodes. However, the central input and output buses still exert some influence on the acoustic field. Therefore, in the final design, only a single ground bus is added (Figs. S7a, d, g), achieving a uniform acoustic wave distribution across the electrodes.

**Fig. S7.** Effects of different ground bus configurations on acoustic waves and electric fields. a-c) Schematic illustrations of different ground bus design approaches. GND: Ground, Vin: Input electrode, Vout: Output electrode. d-f) Electric field distributions for the corresponding ground electrode configurations. g-i) Acoustic wave distributions for the corresponding ground electrode configurations. Note: For computational efficiency, the simulations are performed on the lithium niobate thin-film layer only, without including the silicon substrate.

**6. The reason for the device's tunable mode volume**

The ability to implement larger electrode gaps arises from the properties of 128° Y-cut lithium niobate, which possesses a large piezoelectric stress constant *𝑒_15_* and is relatively insensitive to electrode spacing. Increasing the electrode spacing has only a minor effect on the effective electromechanical coupling coefficient while reducing the impact of parasitic modes, as shown in Fig. S8. Consequently, our HBAR devices can achieve tunable mode volumes while maintaining excellent performance even for large mode volumes.

**Fig. S8.** Simulated conductance response of the resonator with electrode spacings ranging from 120 μm to 820 μm, while the electrode width is kept at 48 μm.

**7.** **CTE Mismatch Reduces *Q_p_* and *Q_s_* in Multiple Overtones**

We provide extended data on the *Q_s_* and *Q_p_* trends across multiple overtones in the Fig. S9. From these observations, we conclude that CTE mismatch may lead to reductions in both *Q_p_* and *Q_s_*.

**Fig. S9.** Admittance spectra of the X-HTBAR measured at temperatures from 50 K to 295 K, corresponding to a–d) different-frequency A1-mode overtones and e–h) A3-mode overtones.

**8. Compensation Layer to Mitigate CTE Mismatch**

To mitigate the impact of CTE mismatch, a SiO_2_ compensation layer can be introduced between the LiNbO_3_ layer and the Si substrate, but its thickness must remain below the acoustic wavelength. If the layer is too thick, it will cause impedance mismatch and reduce the efficiency of acoustic wave injection into the substrate. As shown in Fig. S10a, by introducing a SiO_2_ intermediate layer with finite thickness between the lithium niobate and Si layers, the admittance spectra retain good performance. As illustrated in Fig. S10b, varying the SiO_2_ layer thickness shifts the resonance peak frequencies, and in some cases even enhances the Q factor. As shown in Figs. S10c and 10d, the introduction of a finite-thickness intermediate layer has only a weak impact on acoustic wave injection into the Si substrate. However, the detailed influence of layer thickness, as well as the temperature dependence of device performance with the SiO_2_ layer, requires further investigation.

**Fig. S10.** **a)** Schematic illustration of inserting SiO_2_ intermediate layers with different thicknesses between the lithium niobate layer and the Si layer. **b)** Simulated admittance spectrum of a laterally excited HTBAR with different intermediate-layer thicknesses. **c)** Simulated displacement field profiles of the A1 overtone modes without an inserted SiO_2_ layer. **d)** Simulated displacement field profiles of the A1 overtone modes with a SiO_2_ layer thickness of 1000 nm. The insets show locally enlarged views of the top displacement fields.

**9. Origin of spurious spectral features near the A1 resonance in Fig. 2a**

The additional spectral features observed around the A1 resonance mainly originate from spurious acoustic modes, which are typically higher-order A1 modes (A1-X modes). These modes arise from acoustic reflections and lateral standing-wave formation within the resonator structure, leading to additional resonance peaks in the vicinity of the main A1 resonance, as illustrated in Fig. S11. In contrast, higher-order antisymmetric modes such as A3 are not clearly visible in the impedance spectra because their electromechanical coupling coefficient is significantly smaller than that of the A1 mode, resulting in a much weaker spectral response.

**Fig. S11.** Spurious spectral features induced by higher-order A1 acoustic modes.

**Reference**

1. Wu, J. et al. A new class of high-overtone bulk acoustic resonators using lithium niobate on conductive silicon carbide. *IEEE Electron Device Lett.* **42**, 7, 1061 (2021).
2. Kovacs, G., Anhorn, M., Engan, H. E., Visintini, G. & Ruppel, C. C. W. Improved material constants for LiNbO_3_ and LiTaO_3_. *In Proc. IEEE Int. Ultrason. Symp.* 435–438 (IEEE, 1990).
3. Wong, K. K. Properties of Lithium Niobate. London, U.K.: The Institution of Electrical Engineers, 2002.
4. Nikanorov, S., Burenkov, Y. A. & Stepanov, A. Elastic properties of Si. *Sov. Phys. Solid State* **13**, 2516–2518 (1972).
5. Glassbrenner C. J. & Slack, G. A. Thermal conductivity of silicon and germanium from 3 K to the melting point. *Phys. Rev.* **134**, 1743–1748 (1964).
6. Tolpygo, K. Optical, elastic and piezoelectric properties of ionic and valence crystals with the ZnS type lattice. *Sov. Phys. Solid State* **2**, 2367–2376 (1961).
7. Bougrov, V., Levinshtein, M., Rumyantsev, S. L. & Zubrilov, A. Properties of Advanced Semiconductor Materials GaN, AlN, InN, BN, SiC, SiGe. John Wiley & Sons, 2001.
8. Kamitani, K. et al. The elastic constants of silicon carbide: A Brillouinscattering study of 4H and 6H SiC single crystals. *J. Appl. Phys.* **82**, 3152–3154 (1997).
9. Li Z. & Bradt, R. C. Thermal expansion and thermal expansion anisotropy of SiC polytypes. *J. Amer. Ceram. Soc.* **70**, 445–448 (1987).
10. Gibbs F. & Hill, G. J. The variation of the dielectric constant of diamond with pressure. *Phil. Mag.* **9**, 367–375 (1964).
11. Slack G. A. & Bartram, S. F. Thermal expansion of some diamondlike crystals. *J. Appl. Phys.* **46**, 89–98 (1975).
12. Bernstein, B. T. Elastic constants of synthetic sapphire at 27°C. *J. Appl. Phys.* **34**, 169–172 (1963).
13. Dobrovinskaya, E. R., Lytvynov, L. A. & Pishchik, V. Properties of sapphire. *In Sapphire. Berlin, Germany: Springer*, 55–176, 2009.
14. Bechmann, R. Elastic and piezoelectric constants of alpha-quartz. *Phys. Rev.* **110**, 1060–1061 (1958).
15. Horai, K. I. & Simmons, G. Thermal conductivity of rock-forming minerals. *Earth Planet. Sci. Lett.* **6**, 359–368 (1969).
16. Tomar, M., Gupta, V. & Sreenivas, K. Temperature coefficient of elastic constants of SiO_2_ over-layer on LiNbO_3_ for a temperature stable SAW device. *J. Phys. D, Appl. Phys.* **36**, 1773–1777 (2003).
17. El-Kareh, B. Fundamentals of Semiconductor Processing Technology. Boston, MA, USA: Springer, 1995.
